# AFF3: a new player in maintaining *XIST* monoallelic expression

**DOI:** 10.1093/jmcb/mjy082

**Published:** 2019-01-10

**Authors:** Reiner A Veitia

**Affiliations:** 1 Institut Jacques Monod, Université Paris Diderot, Paris, France; 2 Université Paris-Diderot, Paris, France

**Figure 1:**
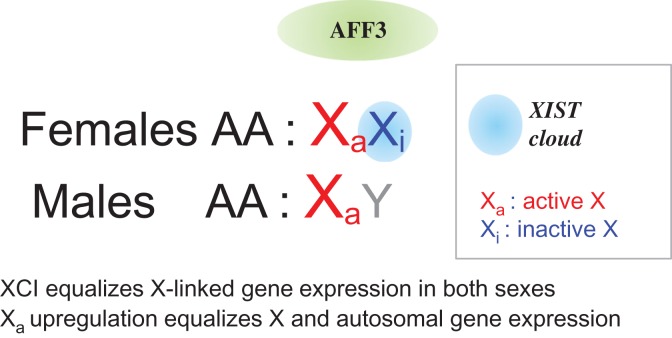
XCI and active X upregulation. XCI equalizes expression of X-linked genes in placental females (XX) and males (XY). Active X (Xa) upregulation should ensure a balance between the expression of autosomal and X-linked gene products. The figure highlights the role of AFF3 in maintaining *XIST* monoallelic expression and hence its potential role in XCI in differentiated cells.

The existence of highly differentiated X and Y chromosomes in mammals raises the issue of X-linked gene dosage compensation between XX females and XY males to avoid what would be similar to a monosomy X in males (Veitia et al., 2015). Inactivation of one copy of the X chromosomes in females provides a dosage-compensation mechanism equalizing the expression of X-linked genes in males and females (Ohno et al., 1959; Lyon, 1961). However, a simple X-chromosome inactivation (XCI) would not ensure a balanced expression between X-linked and autosomal genes (Figure 1). This can be achieved if genes on the active X undergo a two-fold upregulation (Ohno, 1967). Some evidence supports this idea (Veitia et al., 2015 and references therein).

In placental mammals, the X chromosome to be inactivated is randomly chosen during embryonic development and is stably transmitted through cell generations. Early cytogenetic studies in mice harboring X chromosome rearrangements allowed the identification of a region containing a locus necessary and sufficient to trigger XCI when present in two copies. Accordingly, this region was called X inactivation center (*Xic* in mouse, *XIC* in human). In placental mammals, XCI relies on epigenetic factors and is initiated at *XIC/Xic*. An essential player of this process within the *XIC* locus is *X-inactive specific transcript* (*XIST/Xist*) gene, which encodes a long non-coding RNA that is transcribed in a female-specific monoallelic manner. It coats the inactive X, creating an ‘RNA cloud’ (Galupa and Heard, 2015). Although XCI is conserved between mouse and human, divergent strategies are employed to ensure monoallelic *XIST/Xist* expression. For instance, expression of *Xist* in the mouse is tightly regulated and involves random mono-allelic upregulation in peri-implantation stages On the contrary, *XIST* is transcribed and coats both X-chromosomes during early human embryonic development. XCI is achieved following implantation where mono-allelic *XIST* expression is established (Okamoto et al., 2011). This process both in mouse and human must involve a stochastic component to account for the observed random XCI and implies the existence of feedback mechanisms ensuring its maintenance. Mathematical models can help understand the importance of stochasticity and feedback for XCI.

While most molecular studies have focused on the regulation of *XIST/Xist* and the establishment of its mono-allelic expression, much less is known about how this expression status is maintained in differentiated cells. The transcription factor YY1 has been previously shown to be essential for the maintenance of *XIST/Xist* expression in somatic cells (Makhlouf et al., 2014). However, no mechanistic insights are available on the maintenance of the repression of *XIST* on the active X chromosome. This has been addressed by Zhang et al. (2019), in this issue of *JCMB*. These authors have recently shown that AF4/FMR2 family member 3 (AFF3), the central factor of the super elongation complex-like 3 (SEC-L3), is recruited to gamete differentially methylated regions (gDMRs) of imprinted loci and helps regulate their expression (Luo et al., 2016). The same group has now found AFF3 as a new player of monoallelic *XIST* expression maintenance in human cell lines.

The first hint on the involvement of AFF3 in XCI came from the upregulation of *XIST* upon *AFF3* knockdown in human embryonic kidney HEK293T cells according to RNA-seq and quantitative PCR data. Of note, HEK293T cells contain three copies of the X chromosome, two of which are inactive. *In situ* hybridization experiments showed that depletion of *AFF3* led to an increased *XIST* expression and to an increased number of cells with three *XIST* RNA clouds per nucleus (whereas the vast majority of the control cells had the expected two clouds). The same experiment was repeated in lung-derived human IMR-90 cells, which have a normal 46,XX karyotype. As expected, depletion of *AFF3* increased *XIST* expression and led to an increase in the number of cells with two XIST RNA clouds. A possible explanation for the modest increase observed, as noted by the authors, is that besides *XIST* upregulation formation of the clouds requires other epigenetic factors enhancing *XIST* accumulation in *cis* over the chromosome. These results point to AFF3 as a novel repressor of *XIST*.

As the CpG island of the *Xist/XIST* promoter is methylated on the active X chromosome (Heard et al., 1993; other references in Zhang et al., 2019), the authors hypothesized that AFF3 might bind the differentially methylated *XIST* promoter DMR (XIST DMR). Chromatin immunoprecipitation (ChIP-qPCR) experiments confirmed this idea in both IMR-90 and HEK293T cells. Moreover, methylated DNA immunoprecipitation assays after anti-AFF3 ChIP in HEK293T cells showed that AFF3 does bind to the methylated XIST DMR. Bisulfite-sequencing of the XIST DMR after anti-AFF3 ChIP confirmed that it was bound exclusively to the methylated allele. Moreover, treatment of IMR-90 cells with the DNA demethylating agent 5-aza-2′-deoxycytidine (5-aza) increased *XIST* expression, the percentage of cells with two *XIST* clouds and reduced the interaction between AFF3 and the XIST DMR, less methylated after 5-aza treatment.

To obtain further evidence, ENL and AF9, which are also components of the SEC-L3 complex, were also analyzed following the same paradigm. Their depletion upregulated *XIST* and increased the number of cells displaying two XIST RNA clouds in IMR-90 cells. AF9 was also found to occupy the XIST DMR. A series of other experiments showed that the recruitment of AFF3 to the XIST DMR was independent of the molecular platform TRIM28/KAP1. For instance, the occupancy of AFF3 at the XIST DMR was not affected by TRIM28 knockdown. This contrasts with the situation in imprinted loci (Luo et al., 2016). Finally, the knockdown of a series of H3K9 methyltransferases in IMR-90 cells was also tested without any obvious change of XIST expression. This suggests that the H3K9 methylation machineries examined are not major contributors to the maintenance of mono-allelic *XIST* expression in the cell lines studied.

All in all, the results of Zhang et al. (2019) point to a novel role for AFF3 in the regulation of *XIST* monoallelic expression at least in differentiated cells. This study adds another player to the repertoire of factors, RNA and proteins, involved in the complex mechanisms of XCI. *[I thank J.F. Ouimette for his helpful comments on this manuscript.]*
